# Experimental Analysis of Micro-Tribomechanical and Thermal Oxidation Properties of Ceramic-Reinforced Copper-Graphite Composites

**DOI:** 10.1021/acsomega.5c03851

**Published:** 2025-10-21

**Authors:** Esad Kaya, Pelin Çağım Tokat Bi̇rgi̇n, Hediye Aydin

**Affiliations:** † Department of Mechanical Engineering, 53004 Eskişehir Osmangazi University, 26480 Eskişehir, Turkey; ‡ Department of Metallurgy and Material Engineering, 52956 Kütahya Dumlupınar University, 43100 Kütahya, Turkey

## Abstract

Using the powder metallurgy process, this study focused on the impact of varying concentrations of SiC and graphite additions on microstructural, tribomechanical, and thermophysical properties. The resulting composites had a high relative density (>90%). The reinforcement particles exhibited a homogeneous distribution in the copper matrix. No compound developed between the reinforcing and copper phases. There was good interfacial bonding between the graphite and SiC structures. The composites’ overall structural hardness increased by 1.04 to 1.25 times. From 1.13 to 1.40 times, the empirical elasticity modulus increased. With SiC and graphite reinforcement, wear resistance was increased 10-fold to 12-fold for the wear tests. Lean graphite reduced friction by around 2.4 times. The samples that were chosen based on their tribomechanical characteristics underwent oxidation testing. The application of graphite and SiC together resulted in a mere 2.5 times improvement in oxidation resistance, while bare SiC reinforcement enhanced it by almost 30 times compared to pure copper. Lean graphite and graphite added to the structure in combination with SiC had no discernible impact on oxidation resistance. For Cu matrix composites, the operating temperature was critical. Graphite-SiC reinforced samples demonstrated superior properties at temperatures below 300 °C, while lean SiC reinforced samples had superior tribomechanical properties at temperatures over 300 °C.

## Graphical abstract

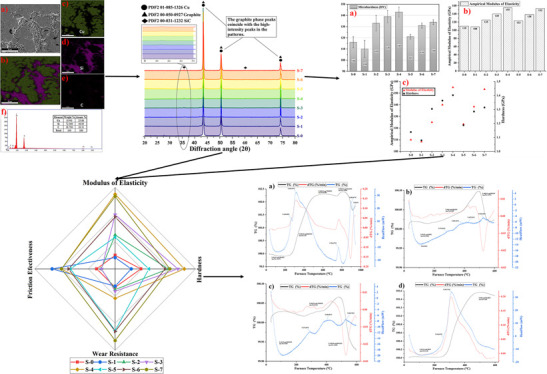

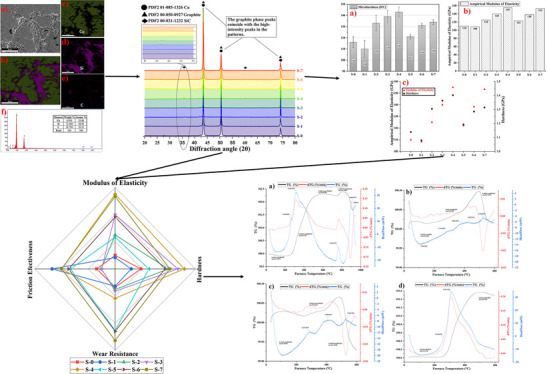

## 1 Introduction

The properties of copper are quite diverse in engineering applications.^1^ The most notable of these are its high electrical conductivity, thermal conductivity, and corrosion resistance.^2^ Additionally, copper can form alloys easily with many metals.^3,4^ Thanks to its high melting point, it is resistant to high temperatures.^5^ This makes it widely used in metallurgy and casting applications.^3^ The limited industrial applications of copper stem from its low wear resistance.^4^ A review of the literature reveals studies investigating the wear behavior of copper-based materials reinforced with oxide and nonoxide ceramic particles.^6–8^ However, there is either no or minimal research on the oxidation resistance of such composites.^7^ Currently, copper-based Metal Matrix Composites (MMC) are preferred over copper and its alloys due to their remarkable structural performance characteristics.^9^ Furthermore, its usage in the industry is steadily increasing due to its enhanced corrosion resistance, thermal properties, and high weldability.^9,10^ These features make copper matrix composites promising and in-demand materials in applications such as heat exchangers, resistance welding electrodes, electrical components, and the automotive industry.^11–13^ Copper matrix composites are generally produced by incorporating finely dispersed ceramic particles into the copper matrix.^13,14^ Dispersion-strengthened copper matrix composites are produced through pressure infiltration, ingot casting, stir casting, and powder metallurgy (PM), wire arc additive manufacturing techniques.^15^ The powder metallurgy method consists of stages such as mixing, compaction, and sintering.^10^ Considering parameters such as the size of the initial powders, the optimum grinding time, and speed, sufficient mixing is very important to obtain a homogeneous mixture with nonagglomerated metal and ceramic particles.^10^ When producing particle-reinforced metal matrix composites using the mixing casting method, certain problems arise, such as difficulties in ensuring the homogeneous distribution of the reinforcement material within the structure.^16^ Poor wetting of the ceramic-based reinforcement material by the matrix material, the formation of pores (porosity) in the internal structure due to trapped gases during mixing, and the occurrence of unwanted chemical reactions due to prolonged contact between liquid metal and reinforcement particles are some of the undesirable situations that may occur.^17^ Hence, given its efficient dispersion of fine particles, the powder metallurgy (PM) method is an excellent choice for producing copper matrix composites.^18^ Cu matrix composite materials are produced using powder metallurgy (PM) at lower temperatures.^13^ As a result, there is less interaction between the matrix and the reinforcing element. The homogeneous distribution of reinforcement elements within the matrix can be achieved through powder metallurgy.^18^ Singh and others synthesized copper matrix composites reinforced with B_4_C, TiC, BN, and chromium using the stir casting method in their study. With an increase in the reinforcement ratio, they obtained materials that were both lighter and exhibited superior mechanical properties compared to pure copper.^19^ Jamwal and colleagues worked on the production of hybrid metal matrix composites consisting of copper, graphite, and silicon carbide using the mixing casting technique.^7^ The increase in graphite ratio has led to a decrease in the hardness of the composites. The wear rate of the composites has been observed to decrease by up to 8% based on the weight of the total reinforcement content.

Previous studies on copper matrix composites have focused on strengthening the Cu phase with various ceramic carbides and oxides.^20,21^ However, these reinforcements are expensive, restricting their large-scale industrial applicability. SiC was deliberately chosen for this study because it offers high performance compared to traditional ceramic additives, along with relatively low cost and wide availability. In addition, while most previous investigations have primarily concentrated on the tribological behavior of Cu-based composites, studies addressing their oxidation resistance remain limited, even though contact-induced oxide formation has a pronounced negative impact on the tribomechanical performance of copper alloys. This study is one of the few systematic investigations of the tribomechanical and oxidation properties of Cu–SiC–graphite composites. The work illustrates substantial enhancements in density, hardness, elastic modulus, and wear resistance of copper by integrating SiC and graphite reinforcements via the powder metallurgy method. It provides new insights into the effect of the type and concentration of the additive on oxidation resistance. It emphasizes the importance of sintering temperature in achieving high relative densities and improved microstructures. This integrated approach, which combines a comprehensive evaluation of the wear and oxidation performance of economically low-cost additives, fills a gap in the literature and highlights the originality and practical importance of the study.

## 2 Materials and Methods

In this study, copper (NaNokar, 99.9%, 44 μm) was used as the matrix, and SiC (SikoUF10, %99,95, 5 μm) and graphite (Alfa Easer, 99.5% purity, 44 μm) were used as reinforcement. The powder mixture was prepared in the ratios specified in Table 1. The powder mixture was homogenized with a Retsch PM 400 planetary mill machine for 12 h and 250 rpm rotation speed parameters. The homogenized powders were formed into 1 cm diameter pellets by applying 6 MPa pressure with a Calver manual press. After 200 MPa pressure was applied to the shaped samples with the cold isostatic press (MSE-CIP), they were placed in alumina crucibles and sintered in a tube furnace (Protherm) at 900 °C in an argon atmosphere for 2 h.

**Table 1:** Stoichiometric Ratios of The Samples

sample code	Cu (vol %)	SiC (vol %)	C (vol %)
S-0	100	0	0
S-1	Bal.	0	2
S-2	Bal.	5	0
S-3	Bal.	10	0
S-4	Bal.	15	0
S-5	Bal.	5	2
S-6	Bal.	10	2
S-7	Bal.	15	2

The composites were molded before characterization, and the sample surfaces were sanded and polished. The density value of the raw powders was measured using a pycnometer, and the theoretical density values of the produced samples were calculated using the simple mixture formula rule. The experimental densities of the samples were calculated using the Archimedes method in accordance with ASTM B962–23. The density of each sample group was measured three times. The average value was reported. Relative densities of the samples were calculated from the ratio of experimental and theoretical densities according to eq 1.^22^

1 $$\rho_{\mathrm{relative}}=\left(\frac{\rho_{\mathrm{experimental}}}{\rho_{\mathrm{theoretical}}}\right)\times 100$$

2 $$E_{\mathrm{Compsite}}=V_{\mathrm{SiC}}.E_{\mathrm{SiC}}+V_{C}.E_{C}+V_{\mathrm{Cu}}.E_{\mathrm{Cu}}$$

The elastic modulus of the samples was also calculated empirically using the rule of mixtures, as shown in eq 2.^22^ Cu, SiC, and C elastic modulus were selected as 110, 410, and 27.6 GPa.^23^ Hardness measurements of the composites were measured using a Shimadzu HMV Vickers microhardness tester according to ASTM E 92 standards.^23^ The average hardness value was calculated by taking five measurements from each sample. The microhardness tests were performed at a load of 25 gf with a dwell time of 10 s. X-ray diffraction (XRD) analysis of the phases of copper matrix composites whose properties were improved with SiC/graphite reinforcements after sintering was performed using PANalytical at Kütahya Dumlupınar University- Advanced Technology Center. During the analysis, an accelerating voltage of 40 kV and a current of 40 mA were applied and characterized by scanning diffraction angles between 20–80°. The microstructures of the composite samples, such as grains, surface morphologies, and distribution of reinforcements in the matrix, were analyzed by elemental mapping to show the homogeneity of reinforcement-matrix distributions and to examine the channel formed by the ball after wear; FEI NovaNanoSEM650 and EDAX Trident energy dispersive X-ray spectrometer (EDS) (Kütahya Dumlupınar University- Advanced Technology Center), and JEOL JSM-5600LV and Hitachi Regulus 8230 model scanning electron microscope (Eskişehir Osmangazi University) SEM were used. The thermal analysis during heating–cooling cycles up to 600 °C of the samples (Cu, %15SiC-Cu, %2 graphite-Cu, and %15SiC/%2 graphite-Cu) prepared to study their oxidation behavior was measured using a dilatometer (Model No. NETZSCH DIL 402C). Sintered pellet samples with a size of 10 mm were used for this purpose. An oxide atmosphere and a 10 °C/min temperature increase were used for the analysis. The produced samples were tested for tribological performance using a CSM tribometer. A 100-m test distance was established, along with a rotation speed of 196 rpm (corresponding to a linear speed of approximately 3 cm/s) and a nominal wear load of 5N applied to a *D* = 3 mm, 100Cr6 hardened bearing steel ball used as an abrasive counter body. The worn surface’s cross-section was measured using a Mitutoyo SJ-400 surface roughness profilometer. The Gaussian filtering technique was used to measure the roughness of the worn surface. Throughout the test, the COF values were noted. The detailed wear mechanism of the produced samples was evaluated using SEM and EDS analysis on the worn surfaces. The obtained results are used to characterize the wear mechanism.

## 3 Results and Discussion

In this study, the theoretical, relative, and experimental densities of copper matrix composites with SiC and SiC/graphite additions were calculated, measured, and given in Figure 1. The relative density of the lean copper sample is the highest value. Due to the low density of graphite, when it is added, the theoretical density decreases, while SiC is added, and the amount of SiC is increased, a similar situation is observed, and the theoretical density values decrease. Compared to S-0, the experimental and relative density decreased, identical to the theoretical density of S-1. The experimental and relative densities of S-2, S-3, and S-4 specimens with SiC addition decreased as the amount of SiC increased. The relative densities of samples S-5 and S-6 with SiC and graphite additions were similar, while S-7 showed a minimal decrease. A minor scratch may have caused the change in this value during the process. In general, the experimental density values of the produced specimens increased/decreased in proportion to the theoretical density values. Since the wetting angles vary with graphite and SiC additives, the relative density of the sample without additives was higher. Relative density values were obtained in the range of 90.9–93.5%. Compared to metal matrix samples produced using pressureless sintering methods, samples with good relative density were obtained. A comprehensive review summarized that the relative density of Cu–SiC composites produced by powder metallurgy methods is generally in the 85–95% range, supporting the values obtained. Additionally, it is reported that the presence of carbon-based phases such as carbon nanotubes or graphite reduces the condensation efficiency of Cu–SiC systems due to their poor wettability with the copper matrix.^24^ Generally, the density values obtained in studies are consistent with the literature and indicate an effective production process.

**Figure 1:**
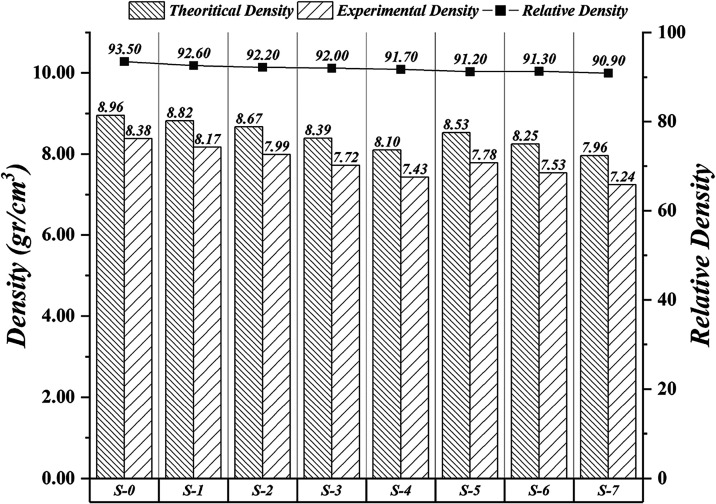
Theoretical experiment and relative densities of samples.

In the micrographs in Figure 2, the light gray areas show the Cu matrix, and the dark gray and angular shapes show the reinforcement component SiC. All images were obtained at 3kx magnification and with a secondary electron detector. Figure 2a shows the sample containing only copper. It is seen that the copper grains are homogeneously distributed. Figure 2b shows the microstructure of the copper matrix composite sample containing 5% SiC by volume. It can be said that SiC particles are homogeneously distributed in the Cu matrix and generally surround Cu particles. This situation is similar for the samples containing 10% SiC by volume, whose microstructure is shown in Figure 2c, and 15% SiC by volume, whose microstructure is shown in Figure 2d. SiC grains are homogeneously distributed in the copper matrix. No agglomeration is observed. SiC grains are also homogeneous in copper matrix composites containing graphite. Figure 2e shows the microstructures of the sample containing only graphite, Figure 2f shows the microstructures of the samples containing 5% SiC, Figure 2g shows 10% SiC, and Figure 2h shows the microstructures of the samples containing 15% SiC and graphite.

**Figure 2:**
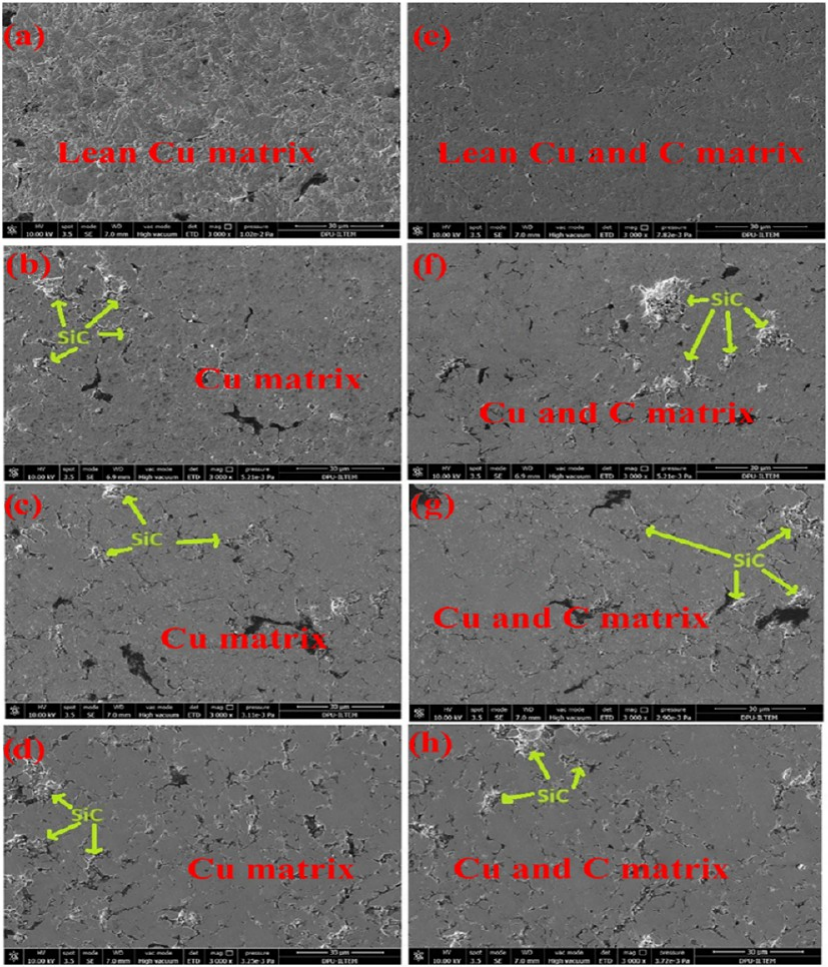
SEM analysis results of the samples: (a) S-0, (b) S-2, (c) S-3, d) S-4, (e) S-1, (f) S-5, (g) S-6, (h) S-7.

Graphite grains are not rigid on the copper matrix, similar to SiC grains. It is dispersed in the copper matrix in an invisible way to the eye. It was detected in EDX analysis. No microstructural differences were observed in the composites’ grain size, boundaries, and porosity with and without graphite. It is essential to ensure a homogeneous distribution of reinforcement in the matrix to increase composite materials’ mechanical, electrical, and thermal properties. SEM studies showed that SiC and graphite-doped composite samples sintered at 900 °C showed a homogeneous and regular distribution. As the SiC addition increased, SiC particles spread like a homogeneous network toward the copper particle boundaries. At high SiC content, SiC particles penetrated the copper particles due to the ductile nature of copper. Minor structural defects (voids, cracks, porosity) were found in the electron microstructures independent of graphite/SiC additives.

The elements and elemental fractions of the samples containing 15% SiC and 15% SiC-2% graphite were analyzed by SEM-EDS and given in Figure 3a,b, respectively. The spot in Figure 3a, which is thought to be a copper grain, was analyzed and named Spot1. In this spot, minimal SiC content was found, and 98.67% copper was detected. For Spot-2, a spot with high SiC content was analyzed. It was mentioned that SiC grains are located at copper grain boundaries. This case is seen in Figure 3a. At Spot-2, which contains 40.36% SiC, it was determined that SiC and copper are intergrown. EDS analysis of the Spot-3 area for Figure 3a indicates that the SiC/Cu balance is evenly distributed throughout the sample. Like Figure 3a, Spot-1 from the copper grain in Figure 3b shows minimal SiC. Unlike Figure 3a, the Si, C, and Cu ratios are slightly higher than in Figure 3a, with the carbon ratios marginally higher due to the effect of graphite addition. In Figure 3a,b, the area results show that the Cu-SiC and Cu-SiC-C ratios are homogeneously distributed throughout the sample.

**Figure 3:**
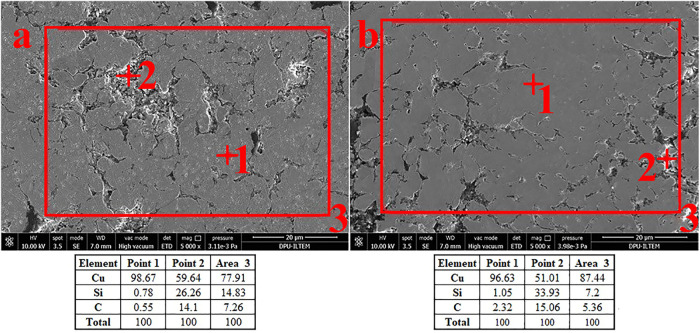
SEM-EDS analysis data of (a) S-4, (b) S-7.

S7, which contains both SiC and graphite, was selected for analysis using the mapping method in Figure 4. In the image taken from a point with high SiC density, the distribution of Si and C elements is relatively uniform over copper. The Si element is represented in purple, the Cu element in army green, and C in orange. It can be seen more clearly in the mapping that the color of the Si element generally separates the color indicating Cu, sometimes like narrow channels, and sometimes like wide channels. The color of C, on the other hand, is mainly seen within the Si grains but is also scattered in spots within the Cu grains.

**Figure 4:**
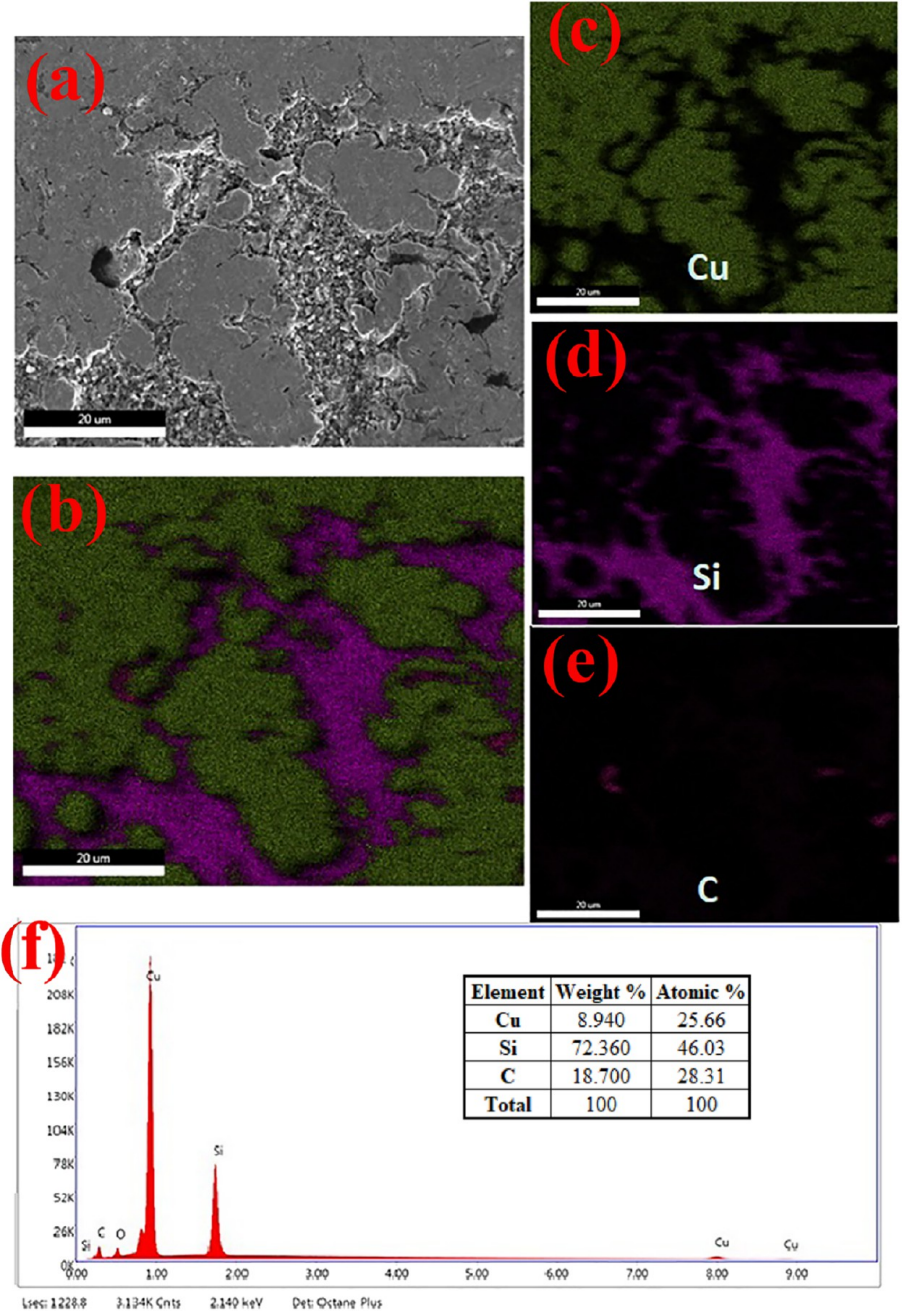
(a) SEM image of the S-7, (b) The total EDS mapping of the sample, (c) The element mapping of Cu, (d) The element mapping of Si, (e) The element mapping of C, (f) The element mapping spectrum of the sample.

XRD analysis was performed to obtain information about the phase contents of SiC and SiC-graphite doped copper matrix composites, and the amounts of these phases. These data are given in Figure 5. The main phase obtained in the composites is the copper phase. The maximum peaks of 2 theta values of the copper element are 43.0, 50.2, and 74.3°. In copper samples with 5% (S-2 and S-5) and 10% (S-3 and S-6) SiC reinforcement, only 100% peak of the SiC phase is minimally detected. In samples with 15% SiC reinforcement (S-4 and S-7), two minimal peaks are observed at 2 theta = 60.0 and 71.2°. No graphite peaks were detected in the graphite-reinforced specimens (S-1, S-5, S-6, and S-7). This situation may be related to the low amount of graphite.

**Figure 5:**
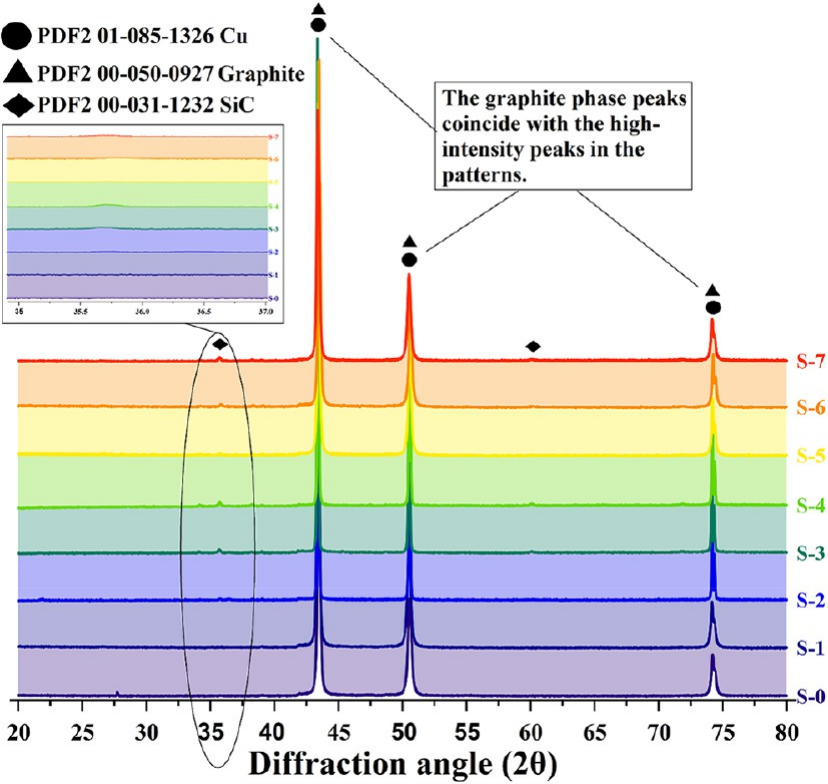
XRD patterns of the samples.

Figure 6a shows the hardness values of pure copper, graphite-doped, SiC-doped, and SiC/graphite-doped copper matrix composites. The hardness value of the S-0 sample without any additives is high compared to the hardness value of the graphite (S-1) doped copper matrix composite sample. While the lubricating properties of graphite increased the wear resistance of the copper matrix, unfortunately, it had a deteriorating effect on the hardness. The decrease in the microhardness value is similar for samples with and without SiC containing the same amount of graphite. The hardness value of the graphite/SiC-doped specimens (S-5, S-6, and S-7) is lower than that of the only SiC-doped specimens (S-2, S-3, and S-4). It is higher than the graphite-doped copper sample (S-1) containing only graphite additives. The hardness change increases significantly when the SiC addition rate increases. The hardness value of the copper matrix composite with 15% SiC (S-4) addition is 1.23 times higher than that of the copper matrix composite without an additive (S-0). The microhardness of the copper matrix composite with 15% SiC and 2% graphite (S-7) addition is 1.22 times higher than that of copper with a nongraphite additive (S-1) and 1.16 times higher than that of copper without an additive (S-0). Although the hardness value of the copper sample produced with graphite doping has decreased the wear resistance, the hardness value has increased compared to the undoped sample (S-0). Incorporating SiC and graphite reinforcements into the copper matrix significantly influenced the mechanical and physical properties of the composites. In the present study, the microhardness of the reinforced composites exhibited a 1.13–1.40 fold increase compared to pure copper, with the most pronounced improvement observed for the hybrid composite containing 15% SiC and 2% graphite (S-7, ∼ 22% increase).

**Figure 6:**
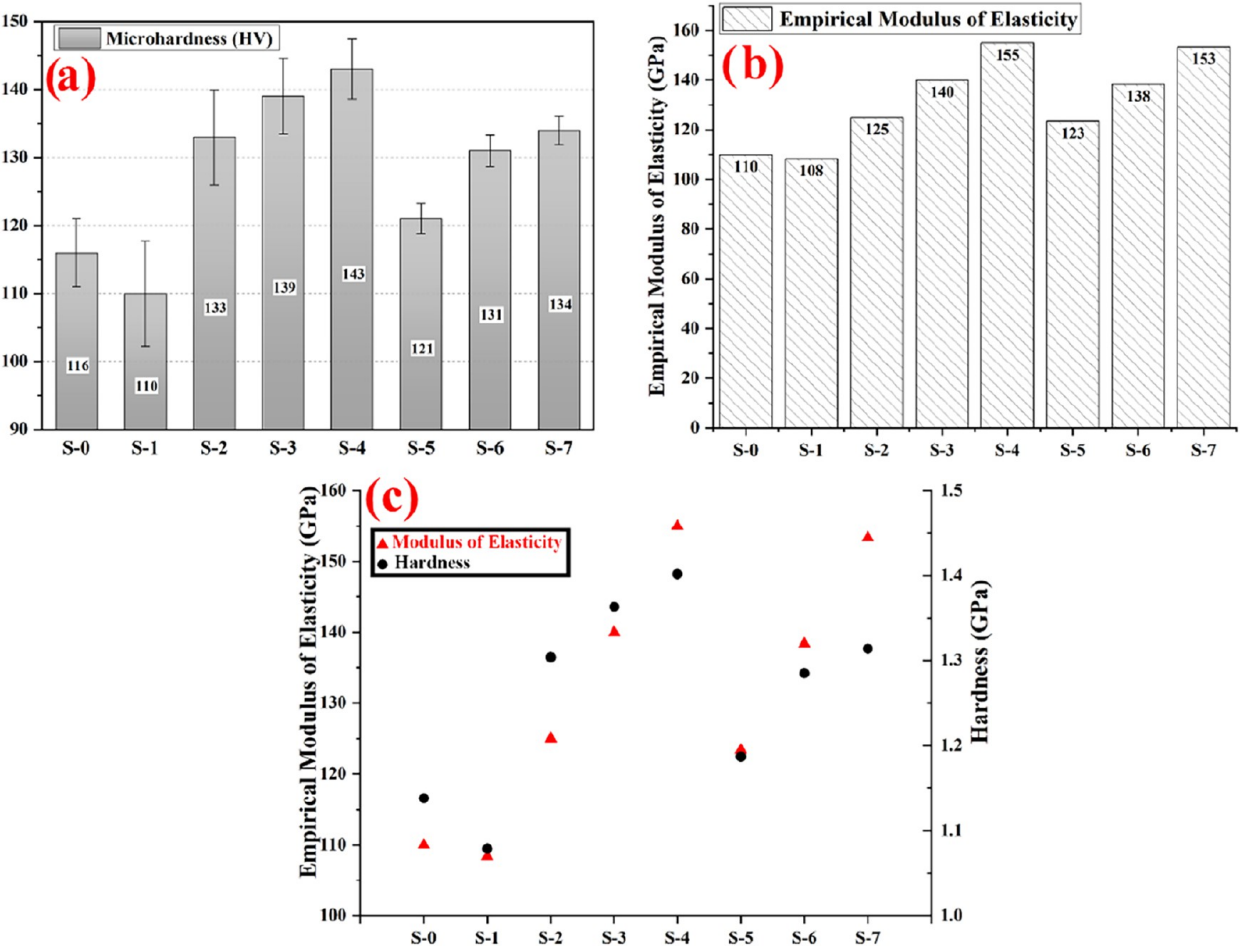
(a) Microhardness of samples, (b) Empirical elastic modulus of the samples, (c) Micromechanical properties comparison of the samples.

Figure 6b shows the empirically calculated elasticity modulus values. As expected, the lowest elasticity modulus value was calculated as 110 GPa in the pure copper sample. The graphite additive added to the structure decreases the elasticity modulus by 1%. The elasticity modulus of graphite is five times lower than that of copper. This decrease in stiffness is expected. The elasticity modulus increases with the SiC ceramic reinforcement added to the structure at different rates. This situation shows that the stiffness in the general structure rises. The elasticity modulus increased between 1.11 and 1.40 times when the SiC was added in different proportions. The stiffness is slightly lower in the SiC and graphite-reinforced samples compared to the samples with plain SiC content. However, it is well-known that graphite has a positive effect on tribological performance. Figure 6c shows the samples’ average hardness and calculated elastic modulus values comparatively. As can be seen, the increase in the elasticity modulus and hardness is correlated. It is observed that graphite addition adds some elasticity to the SiC-containing samples.

Figure 7 shows the samples’ wear rate average and coefficient of friction (COF) diagram. It is observed that the characteristics of wear and friction behaviors seem to be correlated for reinforced samples. Improving the alloy design by integrating lean graphite positively impacts friction behavior, although wear resistance has decreased. As anticipated, the noncarbide doped group (S-1) with a lean graphite content (73.27 × 10^–5^ mm^3^/Nm) had the highest wear rate. This situation is mainly based on the inadequate bearing capacity of nonreinforced copper alloy. Adding graphite without using SiC did not provide sufficient load-carrying capacity to the surface. For only SiC-reinforced groups, the COF increased compared to the nondoped Cu sample (S-0). For the S-4 sample, COF decreased due to a decrease in the contact area. In groups with lean graphite additive content samples (S-1) and all groups with varying carbide and graphite content (S2–S7), wear resistance increased by 1.48 to 12.12 times. It has been observed that including SiC carbide reinforcement in Cu alloys increases their wear resistance. The groups with the highest carbide reinforcement content (S-5, 6, and 7) exhibited the lowest wear rates. Graphite was added to ceramic reinforcing groups to improve friction behavior and wear performance. Together, using graphite with SiC improves friction behavior and wear resistance. It has been demonstrated that the wear resistance of carbide-reinforced samples was increased by 3.65 to 6.75 times by adding the graphite phase.

**Figure 7:**
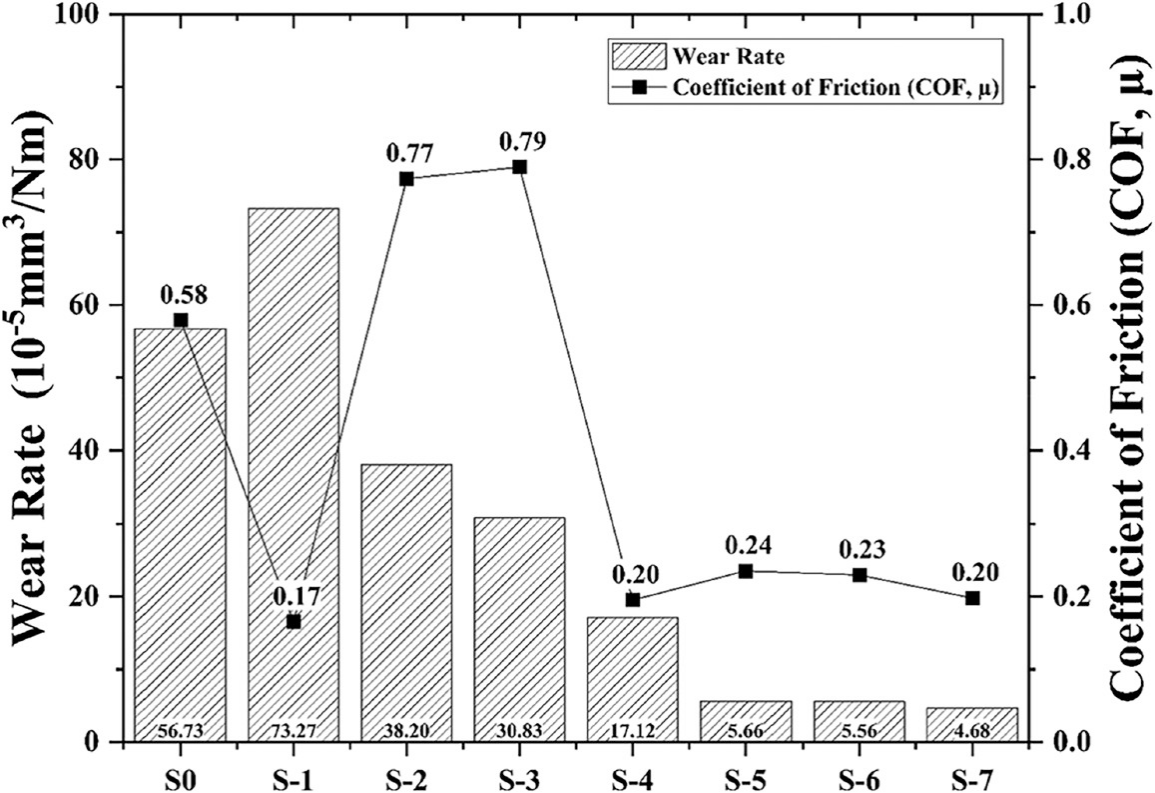
Wear rate and average COF values of the samples.

The friction behavior of samples containing different amounts of ceramic reinforcements and graphite additives is shown in Figure 8. When the friction behaviors are examined in general, the behavior of all samples exhibited the regular regime after the maximum force, which is the static friction point. This case indicates that the tests on all samples are stable. The selected wear load, speed, and distance are ideal for the application. The COF alteration graph of the pure Cu (S-0) and lean graphite added (S-1) samples is given in Figure 8a. With increasing wear distance, the friction behavior of the untreated sample started to waver. Tribochemical oxide structures containing Cu and O formed at the interface damaged the surface. These structures, constantly on the surface, fluctuate the friction behavior. With increasing test distance, these particles moved out of the interface, and the friction behavior stabilized. The friction coefficient has become stable. The friction behavior of the lean graphite added sample (S-1) is seen in Figure 8a. The lubricating effect of graphite is visible. The graphite was thought to move in layers and was plastered onto both surfaces. With increasing wear distance, graphite-containing oxides also formed, resulting in low friction behavior on both surfaces.

**Figure 8:**
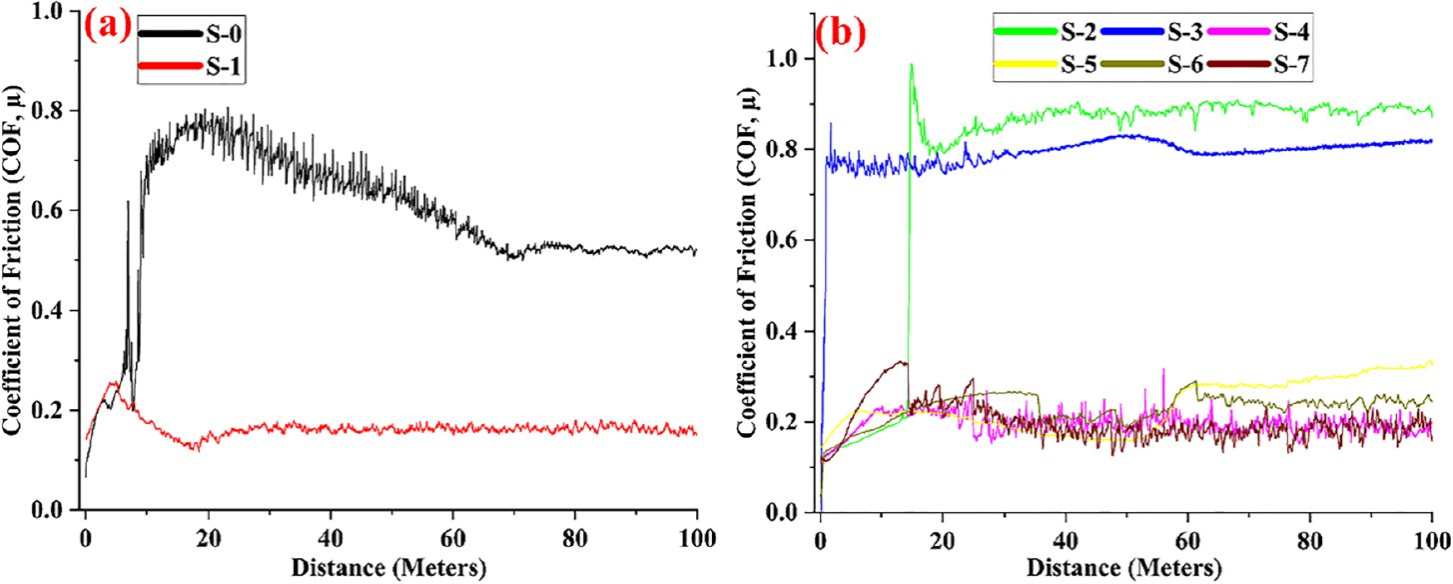
COF variation of the sample with the distance

Figure 8b shows the friction behavior of samples containing different amounts of carbide and graphite. As shown in the figure, the friction behavior of the carbide-doped samples is in the high regime. It is seen that the friction behaviors of pure Cu and lean graphite-added samples are in a higher regime, especially in 5% and 10% carbide-doped samples (S-2 and S-3). The lean carbide contact at these rates could not provide sufficient load-carrying ability to the matrix. It is seen that the friction decreases in the sample (S-4) with a lean 15% carbide content. The increasing carbide reduced the actual contact ratio and decreased the friction coefficient. The friction behavior is clearly in the low regime in the samples containing SiC and graphite additives (S-5, S-6, and S-7). It seems that the effect of graphite causes this situation. Compared to other reinforced samples, a higher friction behavior regime is observed in the sample with a low carbide reinforcement rate (S-5). Even though it contains graphite, the low amount of reinforcement causes metal-to-metal contact and results in low load-carrying ability. It is seen that the friction regime is lower in the samples containing higher carbide reinforcement (S-6 and S-7). Increasing the carbide ratio reduced the hard steel-soft Cu matrix metal contact on the surfaces. The possibility of hard carbide reinforcement and hard steel contact is increased. For this reason, the rising carbide ratio, albeit slightly, caused oscillations in the friction behavior. However, since the upper parts of the carbides on the surface with high hardness were plastered with graphite, the friction decreased due to the laminated movements of graphite layers.

Figure 9 shows the maximum wear channel cross sections measured in the samples. As can be seen, the channels with the largest wear area are the S-0 and S-1 samples. These samples do not contain ceramic reinforcement. A large amount of abraded material accumulation is observed on the surface profile. This occasion is an indication of abrasive wear. When the worn surface profiles of the samples containing plain SiC are examined, it is seen that the volume accumulated on the surface decreases. Especially with the increasing SiC ratio, the surface wear volume decreases. This volume accumulates around the channel and remains in the contact area, causing triple abrasive wear. This fact is the most severe abrasive wear regime. When the three-dimensional profiles of the worn surfaces of the samples containing graphite and SiC are examined, it is seen that the worn volume does not form an accumulation. With the increasing SiC ratio, the peaks of the wear residues on the wear surface decreased. The graphite in the structure also formed layered structures on the surfaces. This situation weakened the abrasive wear.

**Figure 9:**
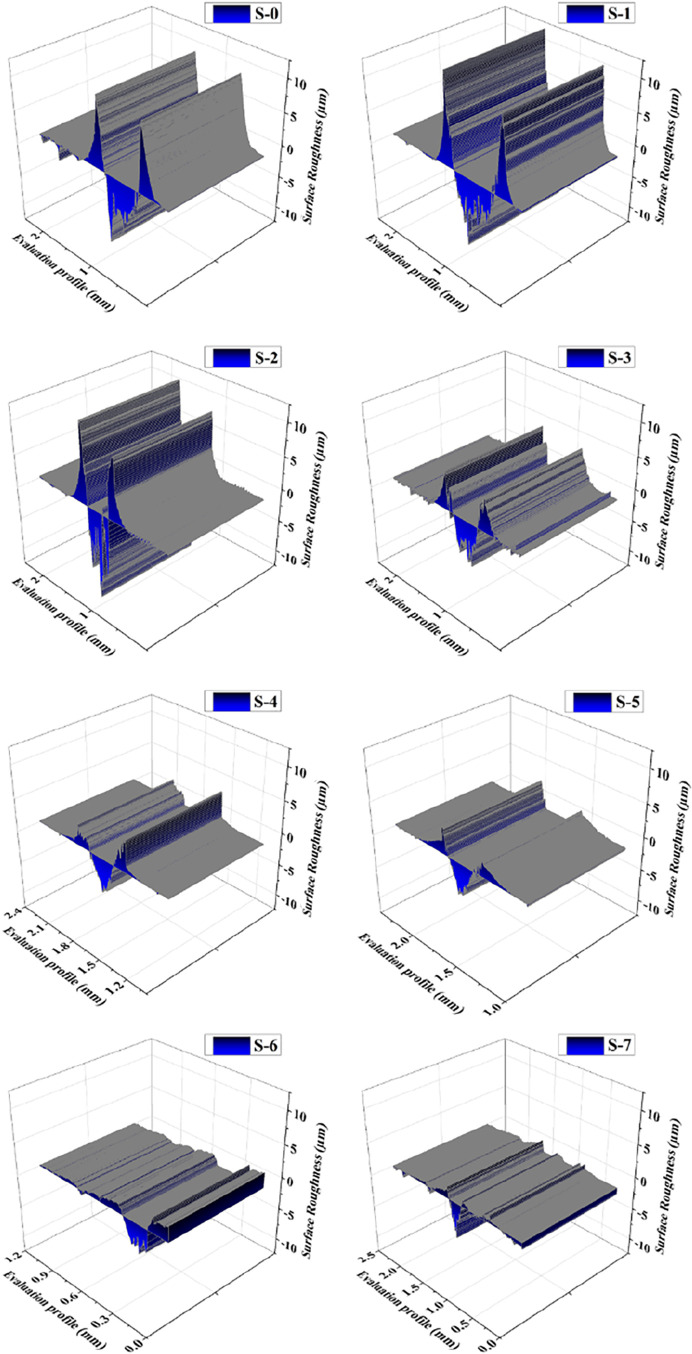
Maximum measured 3d profile of the worn surface of the samples.

Figure 10 shows the SEM-EDS analysis of the worn surfaces of the pure Cu and the lean graphite-added samples. As can be seen, structures with different oxide contents are formed in the pure sample. These oxides appear to contain Cu–Fe (Figure 10a-point-2). It is observed that these oxide structures exhibit low toughness values and tend to break locally. These broken layers exacerbated the wear (Figure 10a-point-1). These broken particles moved away from the contact surface at increasing test distances, and the friction behavior directly stabilized. The worn surface analysis of the lean graphite added sample (S-1) can be seen in Figure 10b. As can be seen, lean graphite additives cannot provide sufficient surface wear resistance. Delamination areas are visible (Figure 10b). These regions contain high amounts of C (Figure 10b points 1, 2, and 3). Graphite-rich Cu-containing layers are delaminated. Although these layers reduce friction by sliding over each other, their wear resistance is low because they have low hardness. It intensified wear by increasing the penetration of the rigid object into the surface, as seen in Figure 10b point 1.

**Figure 10:**
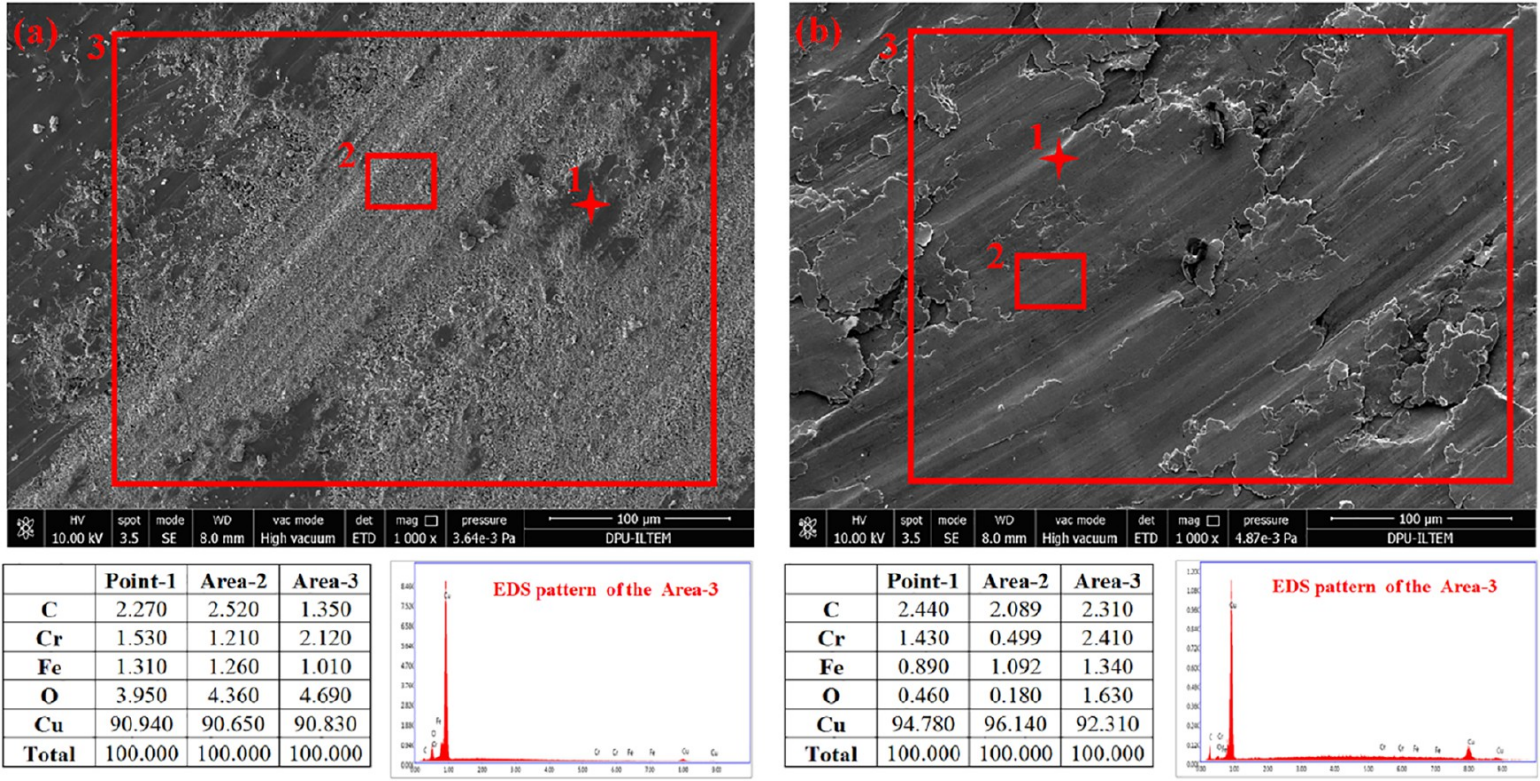
SEM and EDS analysis of the worn surfaces a)­S-0, b)­S-1.

Figure 11 shows the worn surface SEM-EDS analysis of lean carbide-reinforced samples (S-2, S-3, and S-4). Generally, when the wear surface of these three samples is examined, it is seen that the wear mechanism changes with increasing reinforcement ratio. When the surface of the sample (S-2) containing the lowest reinforcement ratio is examined (Figure 11a, point 2), regional oxide formations are observed. The primary dominant mechanism is oxidative. The chemical affinity of the surface is high due to the high concentration of metallic matrix on the surface. These formed oxides broke away from the surface over time and remained at the interface, creating a third-body effect. It indirectly exacerbated wear (Figure 11a, point 1). The surface hardness increased with increasing reinforcement ratio. Figure 11b shows the wear surface of sample S-3 containing 10% SiC reinforcement. As seen at Point 1, Cr-containing fragments broken off from the opposite object were detected. Increasing the SiC reinforcement phase ratio on the sample surface increased its strength. The wear behavior of this sample was partly oxidative and partly abrasive. When the generally worn surface of the sample (S-4) belonging to the highest reinforcement ratio is examined, it is seen that the dominant wear mechanism is entirely abrasive. Scratching can cause damage to the surface. A high amount of Cr was detected on the surface of this sample, which has the highest hardness value (Figure 11c, area 3). Thanks to its high rigidity, hard particles can be seen breaking away from the opposing object. The ruptured hard particles were broken into pieces where they contacted the stiff reinforcement and did not create an agglomerate effect. There are no signs of oxidation in the system. Formed third body wear debris stuck on the surfaces could not form agglomerations.

**Figure 11:**
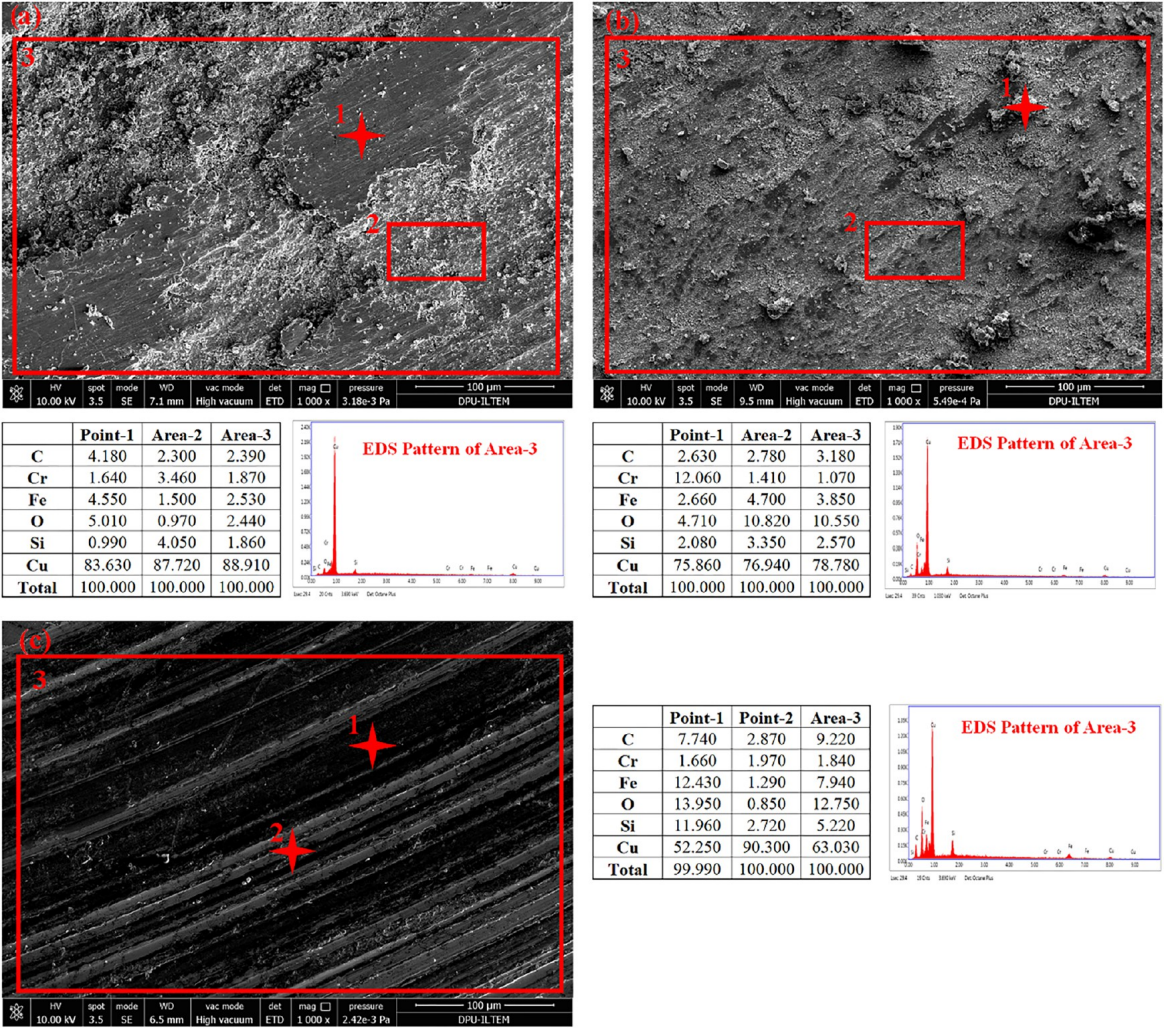
SEM and EDS analysis of the worn surfaces: (a) S-2, (b) S-3, (c) S-4.

Figure 12 shows the worn surface analysis of graphite-containing and carbide-reinforced samples. The effect of the graphite additive is seen to a great extent. Oxidative and abrasive effects on the surfaces are eliminated. When the wear surface of the sample with a low reinforcement ratio (S-5) is examined, abrasive and oxidative effects are visible, albeit partially (Figure 12a, points 1–2). These shiny objects visible on the surface are structures composed of Fe–Cr–Cu elements. They remained at the interface, causing wear to be aggravated. It is seen that the formation of these structures decreases with increasing reinforcement. The increasing reinforcement allowed contact between the hard steel ball and the hard SiC particles dispersed on the surface. The graphite in the structure was delaminated and plastered on both surfaces. The wear severity decreased with the increase in reinforcement ratio. This mechanism was observed primarily in graphite-added 10 and 15% SiC-reinforced samples (S-6 and S-7). (Figure 12a,b). When the worn surface of the S-7 sample, which has the highest reinforcement ratio, is examined, oxidative and abrasive effects are not observed. The high reinforcement ratio and graphite additive protected the surface from wear and friction.

**Figure 12:**
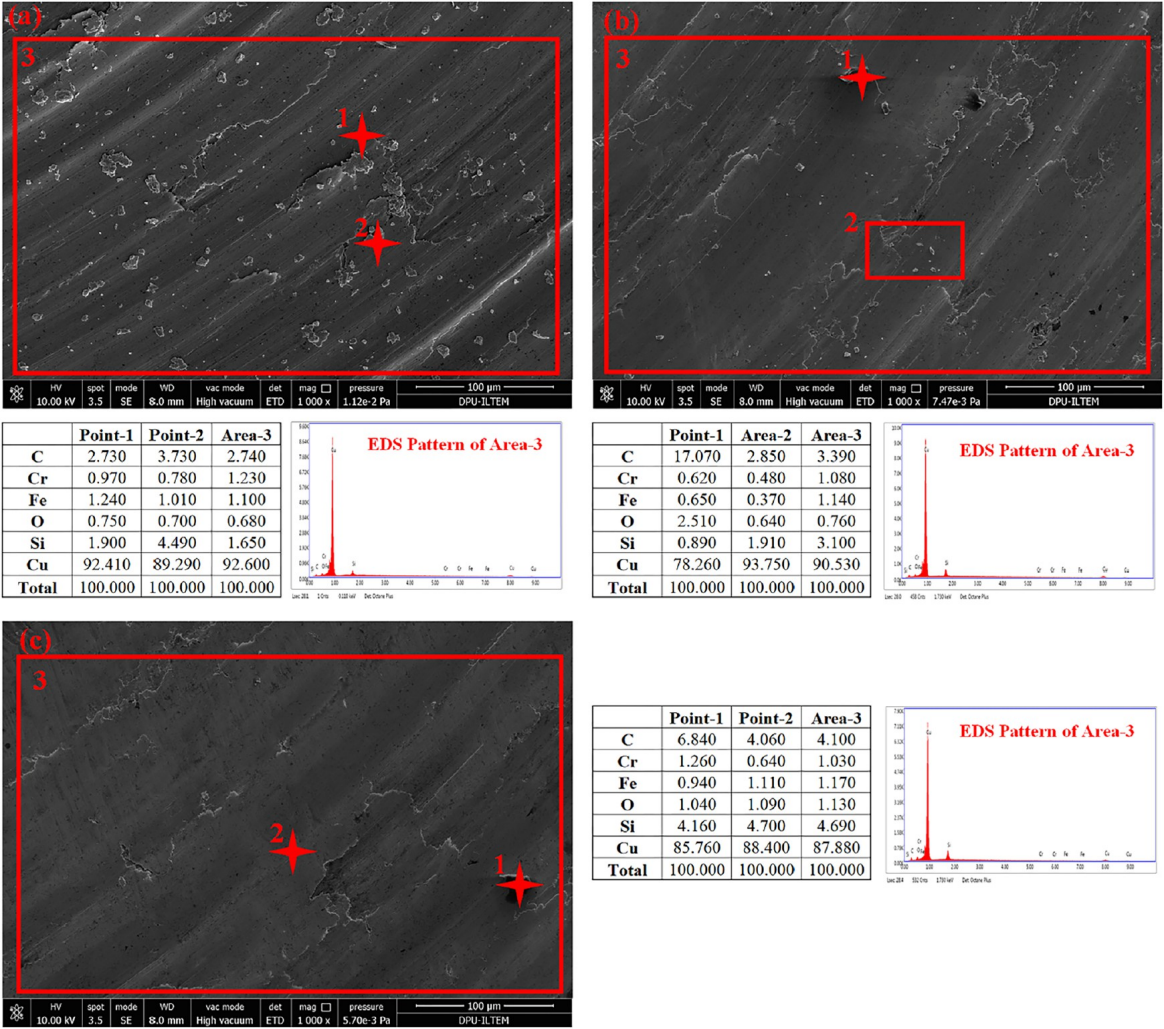
SEM and EDS analysis of the worn surfaces (a) S-5, (b) S-6, (c) S-7.

Figure 13 shows the comparative tribological and mechanical properties. As can be seen from the calculations, the highest elasticity modulus values were seen in samples with bare SiC content. The S-4 and S-7 samples exhibited the highest stiffness. Similarly, samples with bare SiC addition also show superior hardness behaviors. When the samples are examined in terms of tribological behavior, the addition of SiC dramatically improves the wear resistance of the samples. The highest wear resistances were seen in samples with the highest SiC content. In addition, graphite addition dramatically improves the friction behavior of the samples. The lowest friction is seen in the sample with bare graphite addition. The addition of graphite to the structure, together with SiC, dramatically improves wear resistance performance. While chemical compositions with bare graphite addition can be considered for areas where friction is directly essential, it is thought that chemical compositions with high SiC addition can be selected in areas where wear resistance is also essential. As can be seen, tribo-mechanical performance is very effective in determining the optimum chemical composition. Therefore, the oxidation behavior of the bare graphite doped sample (S-1) and the samples (S-4, S-7) exhibiting superior tribomechanical performance were characterized.

**Figure 13:**
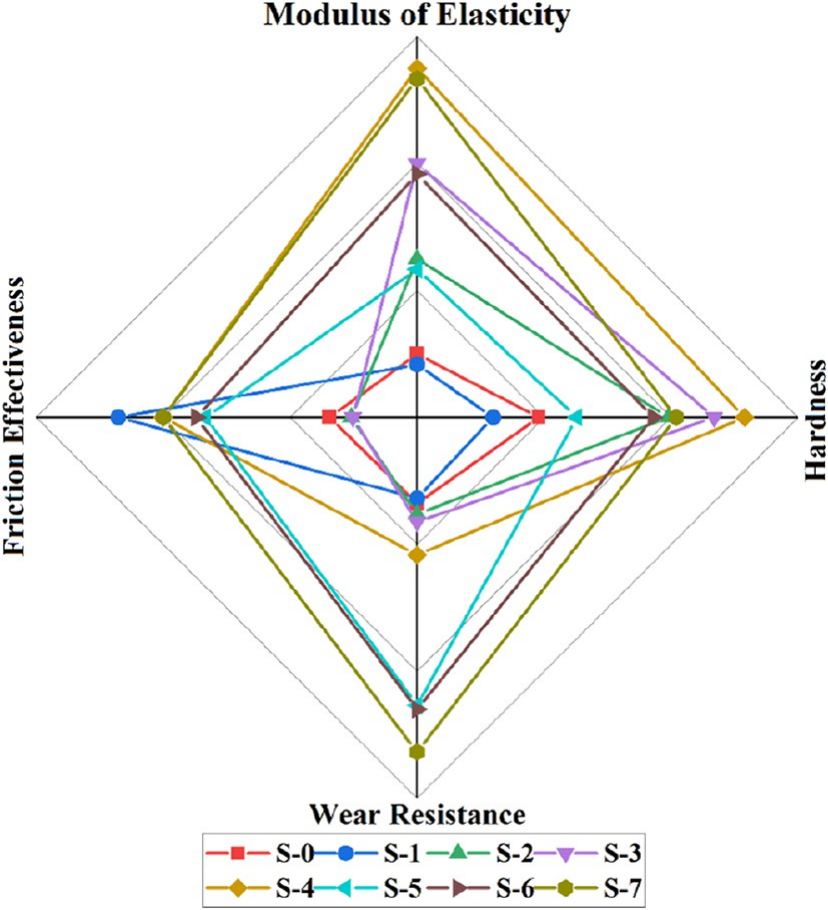
Relative tribo-mechanical properties comparison of the samples.

Thermal analyses provide information on physical phenomena, such as phase transitions and oxidation, as well as chemical reactions, including thermal decomposition. Figure 14a shows TG and dTG curves of pure copper (S-0), Figure 14b shows copper with 2% graphite (S-1), 14-c shows copper with 15% SiC (S-4), and Figure 14d shows TG and dTG curves of copper matrix composites containing 15% SiC-2% graphite respectively (S-7). The samples were heat-treated to 600 °C using a heating rate of 10 °C/min. A literature review did not reveal any studies on the oxidation behavior of copper matrix composites obtained by the powder metallurgy method, thereby enhancing the originality of the current study. Narciso and colleagues^25^ produced SiC-reinforced Cu–Si composites via the pressure infiltration method and investigated the composites’ thermal conductivity and thermal expansion. No significant difference was observed between the samples when the specific gravity changes were examined after thermal analysis. The addition of SiC was found to have minimal effect on the oxidation resistance of the composite samples. The test temperature was set at 600 °C based on information that the oxidation rate of copper is relatively high even at relatively low temperatures.^5^ The chosen test temperatures were as copper does not oxidize significantly below 300 °C.^26^ The graph in Figure 14a shows a weight gain of approximately 2.39% up to 600 °C for the pure copper. The graph in Figure 14b shows a total mass increase of roughly 0.087% up to 488 °C. The reason for this is thought to be the oxidation of the copper matrix in the experiment, which was performed in an oxygen environment, causing an increase in mass by retaining oxygen on its surface. When Figure 14a and c are evaluated together, the mass increase of the sample produced with 15% SiC addition was 30 times less than that of pure copper and equivalent to the lean graphite doped sample (S-1). In other words, it can be said that the oxidation resistance of the SiC-doped sample (S-4) is 25 times better compared to the pure copper sample (S-0). The oxidation data of the samples containing 2% graphite (S-1 and S-7) are given in Figure 14b,d. Around 600 °C, some weight gain was observed in both graphs. The reason for the weight gain is that the graphite in the sample is transformed into oxide form and retains oxygen in the sample.

**Figure 14:**
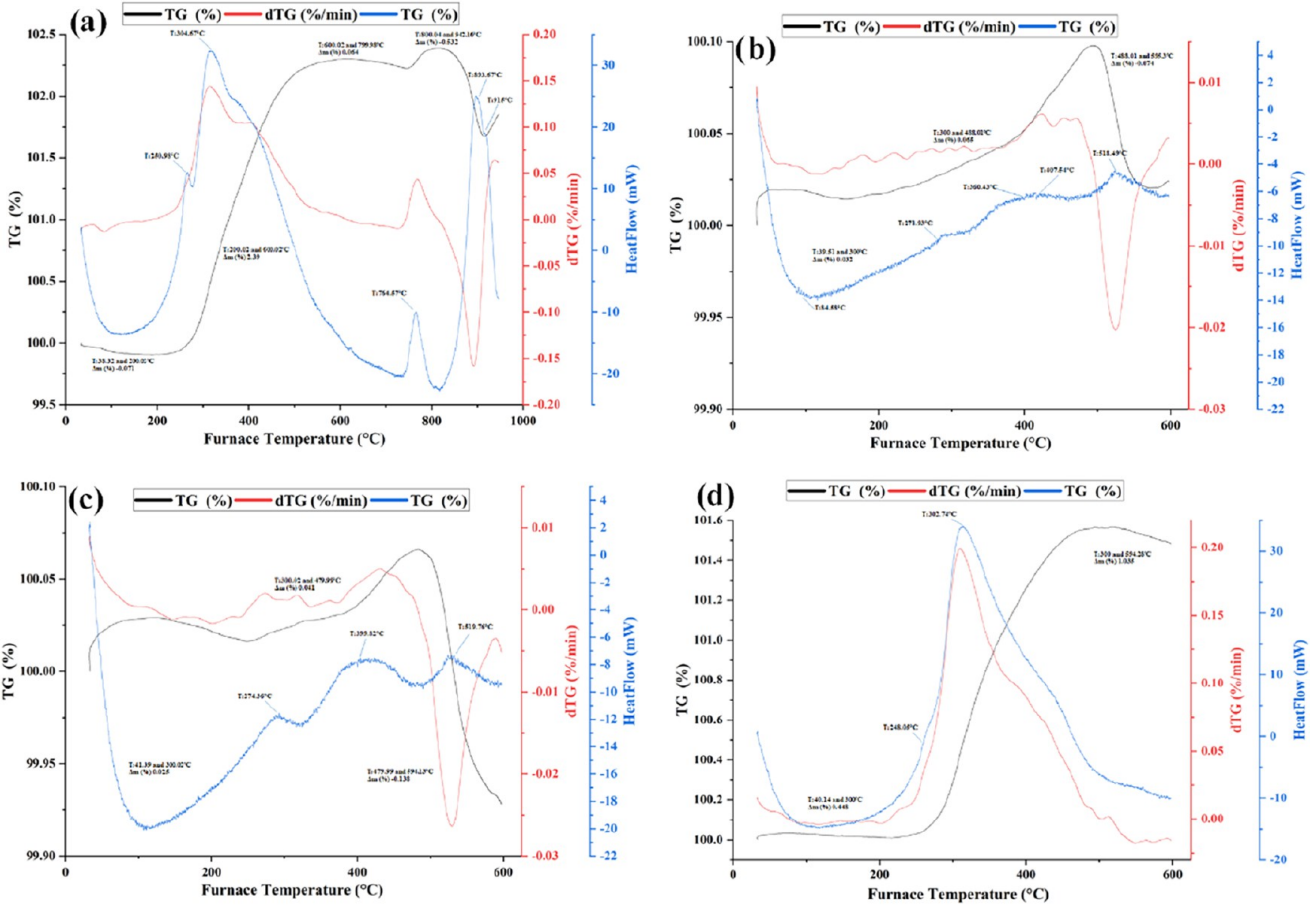
Thermal analysis curves of mechanically optimally selected samples: (a) S-0, (b) S-1, (c) S-4, and (d) S-7.

Figure 15 shows the weight losses and gains at the end of the oxidation test in the samples selected as optimal in tribomechanical properties. The highest weight gain is seen in the pure copper sample. The S-4 sample containing lean SiC reinforcement exhibited weight loss. Weight gain was observed in the graphite-doped samples (S-1 and S-7). The second-highest mass gain was determined in the S-7 sample. This sample contains high amounts of SiC and graphite additives. Although it exhibits good tribological properties, its thermal stability is lower than that of the plain SiC-doped sample. It is seen that the graphite solid has a deteriorating effect on the oxidation resistance. Oxidation tests were performed at 600 °C. It was observed that the graphite additive did not give effective results for mechanical components that will operate at temperatures close to 600 °C.

**Figure 15:**
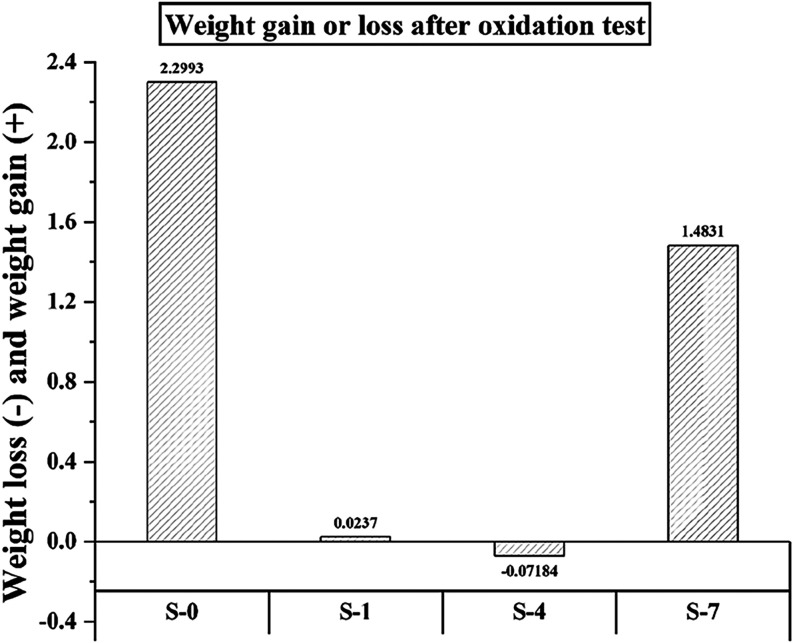
Weight loss and gain of the samples after the oxidation test.

Figure 16 shows a comparison of the studies conducted in the literature and the current study. A review of the literature reveals that the production of copper matrix composite samples or coatings has been extensively studied using different reinforcement phases. Similar hardness results have been obtained in the literature for Cu matrix composites and coatings produced with different reinforcement materials.^5,13,27–30^

**Figure 16:**
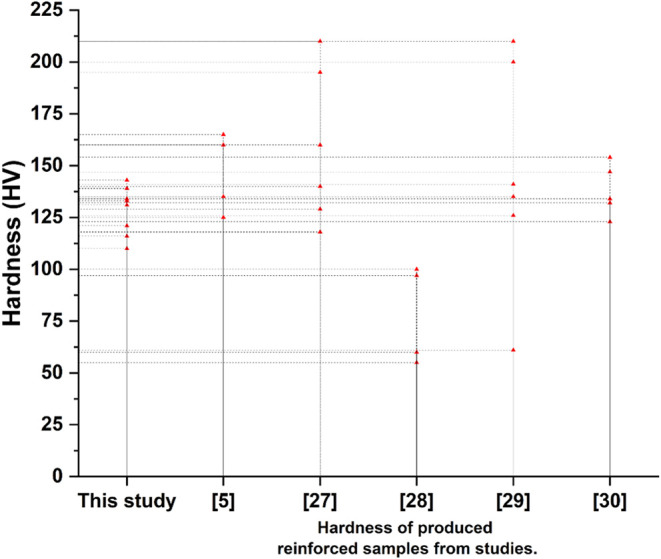
Comparative mechanical properties results of the current studies.

## 4 Conclusion

The present study synthesized Cu matrix composites reinforced with graphite and SiC using the powder metallurgy method. The composites’ microstructural, mechanical, tribological, and thermophysical behaviors were evaluated. The obtained results were summarized as follows.

- Composites had a relative density of 90.9–93.5%, decreasing with greater reinforcement ratios due to lower ceramic particle density. SiC and graphite were evenly distributed in the copper matrix microstructurally, with no intermediary compounds. EDS and mapping verified the reinforcing elemental composition and homogeneous dispersion. No intermediate chemicals were found in the phase analysis of Cu, SiC, and graphite. Low graphite content caused weak SiC peaks and overlapped graphite–Cu peaks.
- The highest microhardness was obtained in the composite with 15% SiC and 2% graphite, showing about 1.2 times improvement over pure copper. In general, the composites exhibited 1.1–1.4 times higher hardness than pure copper. The lowest elastic modulus was 110 GPa in pure copper. Graphite caused a slight decrease, while SiC additions increased the modulus by about 1.1–1.4 times.
- Graphite and carbide reinforcements improved the tribological properties of copper alloys. The wear resistance of the sample containing a low amount of graphite is lower. The SiC reinforcement has increased the wear resistance by a factor of 3.3. The wear resistance of the sample containing SiC and graphite has increased up to 12 times compared to pure copper. The addition of graphite has reduced the friction coefficient by approximately 2.4 to 2.9 times.
- The thermal oxidation test on samples selected based on tribomechanical characteristics showed that carbide reinforcement increased oxidation resistance by 2–30. Pure SiC addition increased oxidation resistance, but graphite or graphite plus SiC decreased it. The 15% SiC-reinforced graphite composite S-7 sample exhibited optimum tribological performance for machine parts at high sliding speeds and loads. The lean 15% SiC reinforced chemical composition is ideal for components operating at temperatures over 300 °C, including tribo-mechanical loads.

## Data Availability

Data used is available throughout the manuscript text.
